# Late Migration of Covered Metal Stent to the Stomach Through a Spontaneous Choledochoduodenal Fistula in a Patient With Malignant Biliary Obstruction

**DOI:** 10.4021/gr452w

**Published:** 2012-05-20

**Authors:** Yoshiki Katakura, Tsutoshi Asaki, Seitaro Adachi, Ikuma Yasuda, Michifumi Toyomizu, Yosho Fukita

**Affiliations:** a Department of Gastroenterology, Seirei Yokohama Hospital, Yokohama, Japan

**Keywords:** Covered biliary metal stent, Choledochoduodenal fistula, Stent migration, Malignant biliary obstruction

## Abstract

We report a case in which a spontaneous choledochoduodenal fistula occurred after biliary covered self-expanding metal stent (SEMS) placement and a late transfistula migration of the stent in a patient with malignant distal biliary obstruction. A partially covered WallFlex biliary stent (Boston Scientific) was appropriately implanted in the common bile duct. Subsequently the patient received chemotherapy with gemcitabine. After 7 months of the SEMS insertion, the patient presented with frequent vomiting. Abdominal computed tomography revealed the obstruction of the duodenal descending part and the migrated stent in the stomach. A choledochoduodenal fistula was observed endoscopically at the proximal point of the duodenal obstruction. These findings can cleanly account for the SEMS migration through the fistula. The mechanism of formation of the fistula is mostly associated with a mechanical contact between the bile duct wall and the SEMS edge, which is pushed up in the direction of the duodenum because of the enlargement of the primary tumor, finally penetrating through the duodenal wall. To our knowledge, this is an extreme unusual case, which has been unreported previously. Therefore, we emphasize the necessity of being alert to the potential for such complications in cases involving placement of SEMS for malignant biliary obstruction.

## Introduction

The placement of self-expanding metal stents (SEMS) is widely accepted for palliative treatment of patients with unresectable malignant distal biliary obstruction because these stents are patent for a long period of time [1, 2]. Although covered SEMS have been recently developed to prevent tumor ingrowth and maintain stent patency [3, 4], complications such as stent migration, cholangitis, cholecystitis, and pancreatitis have simultaneously been reported with these stents [4]. The rate of stent migration, in particular, is 1.8% to 8.3% [3-7]. Spontaneous choledochoduodenal fistulas are rarely associated with the placement of SEMS [8-10]. To our knowledge, this is the first report on late migration of a covered SEMS to the stomach through a spontaneous choledochoduodenal fistula in a patient with malignant biliary obstruction.

## Case Report

**Figure 1:**
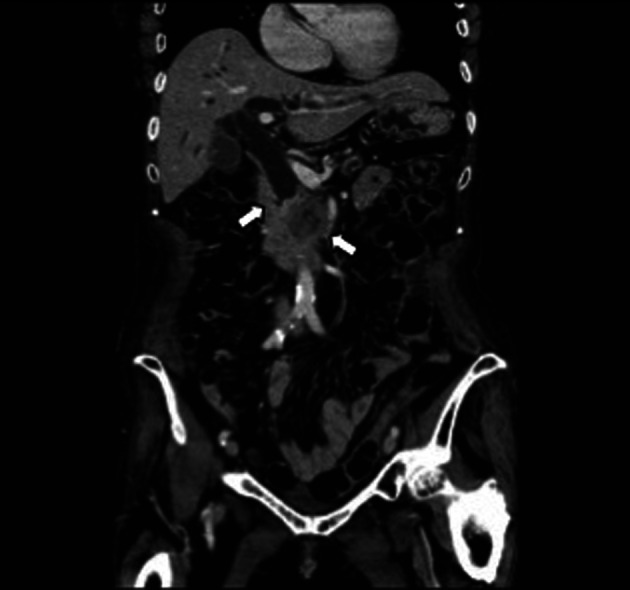
Abdominal CT revealed distal biliary obstruction, and a lesion approximately 3 cm diameter mass lesion located at the head of the pancreas at the first diagnosis (arrow).

**Figure 2:**
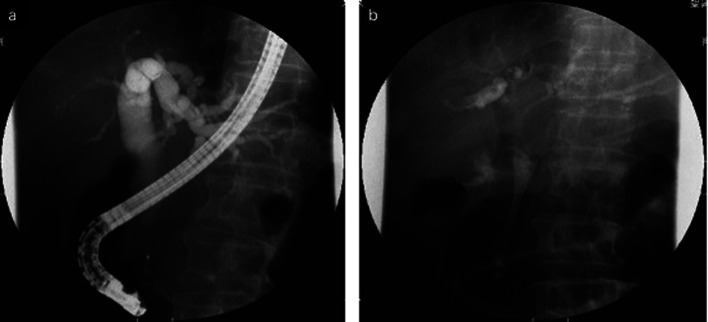
a: ERCP revealed an obstruction at the distal portion of the CBD. b: A partially covered WallFlex biliary stent was appropriately implanted in the CBD.

A 79-year-old woman sought medical advice at a nearby clinic with the chief concern of jaundice. Abdominal computed tomography (CT) revealed dilatation of the biliary tree, the common bile duct (CBD), and a lesion approximately 3 cm in diameter located at the head of the pancreas (Fig. 1). Levels of carbohydrate antigen 19 - 9 (CA19 - 9) and total bilirubin (TB) increased to 1200 U/mL (reference range, < 37 U/mL) and 12.8 mg/dL (reference range, < 1.2 mg/dL), respectively. The patient underwent an endoscopic retrograde cholangiopancreatography (ERCP), which revealed an obstruction at the distal portion of the CBD (Fig. 2a). Pancreatic adenocarcinoma was diagnosed on the basis of the results of cytological examination of the bile. Subsequently, successful endoscopic drainage was performed with the placement of a 7-Fr Zimmon Biliary Stent (Cook Medical, Bloomington, IN, USA). A SEMS was inserted after 7 days; a WallFlex Biliary RX Partially Covered (Boston Scientific, Watertown, MA, USA) stent measuring 10 x 40 mm was inserted because the patient refused to undergo surgical resection (Fig. 2b).

After all therapeutic options were discussed with the patient, she decided to receive systemic chemotherapy with gemcitabine (GEM). GEM was administered weekly at a dose of 1000 mg/m^2^ as a 30 min intravenous infusion for 3 consecutive weeks, followed by a 1 week rest. GEM administration was started 3 weeks after insertion of the metal stent, and 5 courses were administered. The levels of CA 19 - 9 continued to decrease at 3 courses of GEM. Remission was evaluated as stable disease on abdominal CT scan at least until 4 courses of GEM therapy. Abdominal CT at the end of the fourth course of GEM-therapy revealed the SEMS in the CBD. The TB levels decreased immediately and normalized over a month after the first biliary stenting up to the completion of therapy.

**Figure 3:**
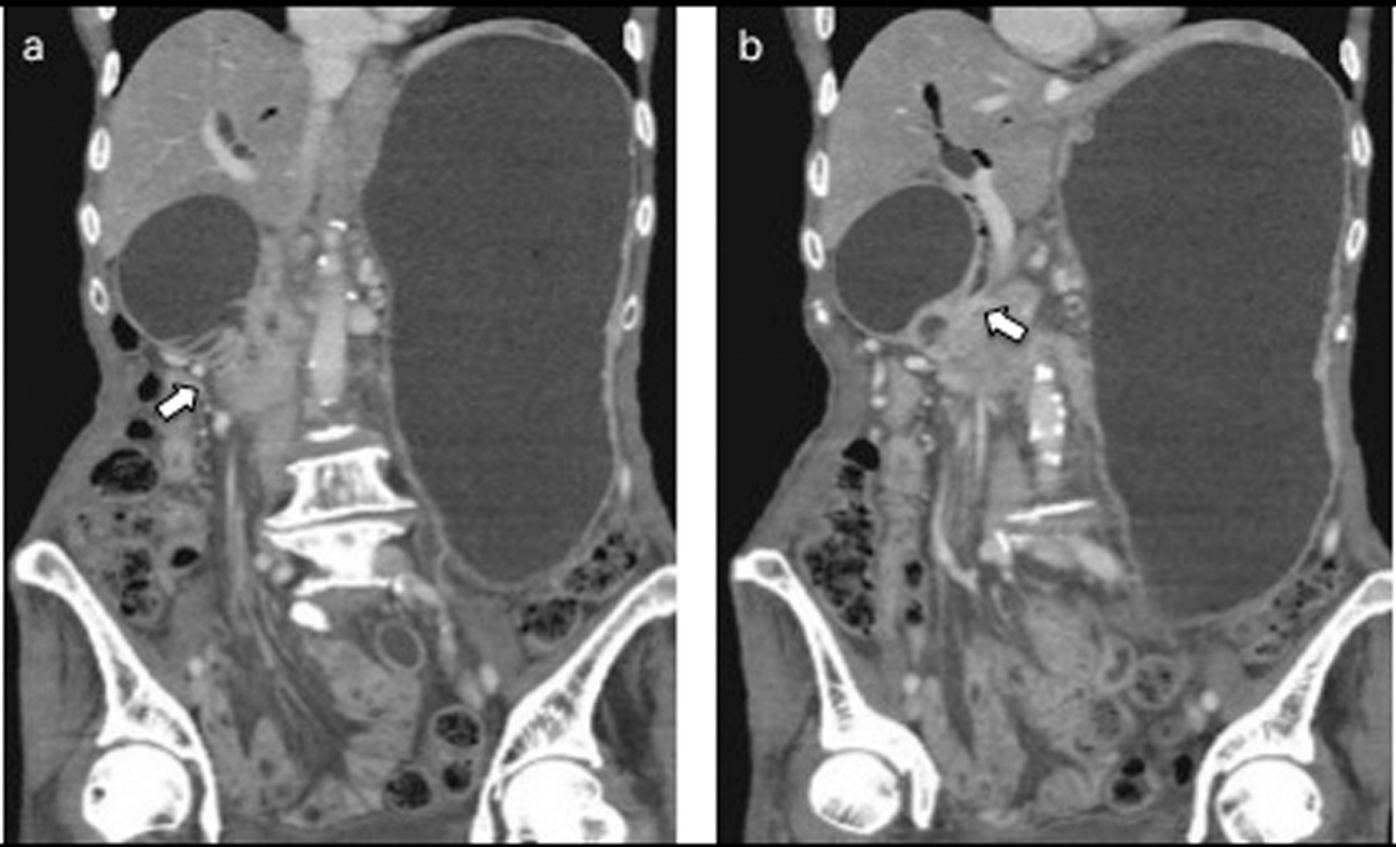
a: Abdominal CT after 7 months of insertion of the metal stent revealed tumor growth in the pancreas and marked distension of the stomach and an obstruction of the descending part of the duodenum by the tumor (arrow). b: The SEMS was not present in the CBD (arrow).

**Figure 4:**
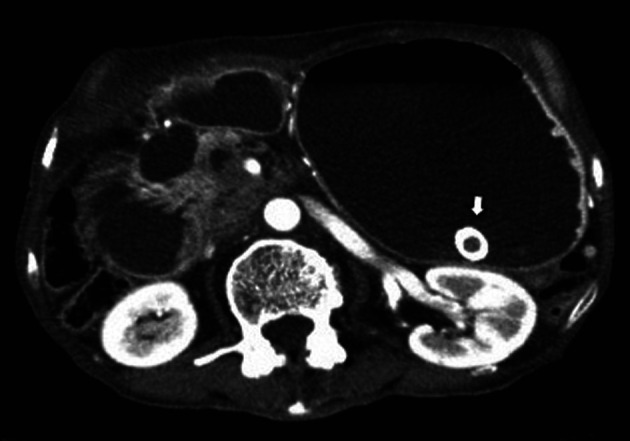
Abdominal CT showed the presence of the SEMS in the stomach (arrow).

**Figure 5:**
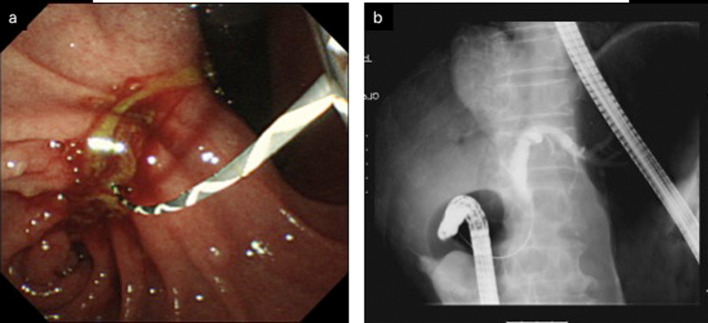
a: Duodenoscopy disclosed an obstruction of the descending part of the duodenum and a choledochoduodenal fistula at the proximal point of the duodenal obstruction. b: Cholangiogram was demonstrated by introducing contrast medium into the fistula.

**Figure 6:**
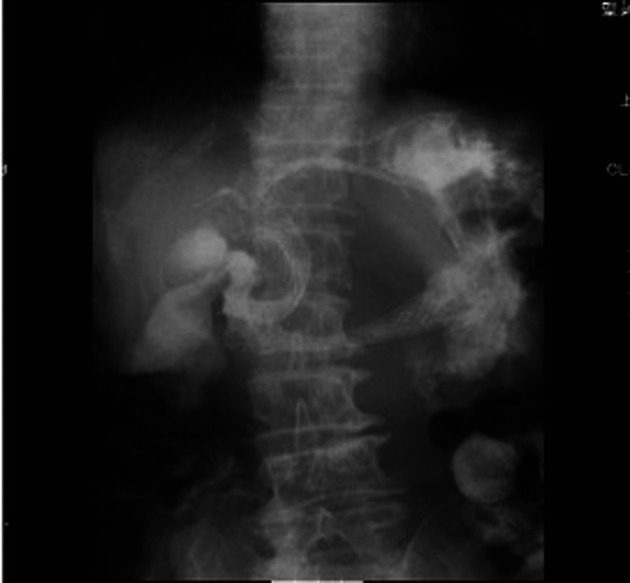
A duodenal WallFlex stent was inserted into the duodenum, and a fully covered WallFlex biliary stent was also inserted into the choledochoduodenal fistula, respectively.

After 7 months of insertion of the metal stent, the patient presented with frequent vomiting. Abdominal CT revealed tumor growth in the pancreas and marked distension of the stomach (Fig. 3a, b). The descending part of the duodenum was obstructed by the tumor (Fig. 3a). The SEMS was not present in the CBD (Fig. 3b). The SEMS was observed in the stomach (Fig. 4). Duodenoscopy revealed an obstruction in the descending part of the duodenum and a biliary outflow from the proximal point of the duodenal obstruction. A choledochoduodenal fistula was diagnosed by introducing contrast medium into the outlet of bile (Fig. 5a, b). Finally, we inserted a WallFlex Duodenal (Boston Scientific, Watertown, MA, USA) stent measuring 22 x 60 mm into the duodenum; in addition, we inserted a WallFlex Biliary RX Fully Covered (Boston Scientific, Watertown, MA, USA) stent measuring 10 x 60 mm into the choledochoduodenal fistula (Fig. 6). Subsequently, the patient could be fed orally, but she died of peritonitis carcinomatosa 2 months after duodenal stenting. Obstruction of the duodenum and jaundice did not recur till the death of the patient.

## Discussion

Nowadays, placement of SEMS has become the standard method for treatment of malignant distal biliary obstruction [1, 2]. Histological examination indicates that the metallic mesh is embedded in the wall of the bile duct several days after stent placement. This mechanism is based on mucosal hyperplasia between the stent struts and complete integration of the stent in the normal bile duct epithelium, as well as in the ingrowing neoplastic tissue is achieved [11, 12].

To increase stent patency, covered metal stents have been developed with the main aim of preventing tumor ingrowth [3, 4]. In a prospective randomized study by Isayama et al., 112 patients with distal malignant biliary obstruction treated by endoscopic insertion of a polyurethane (manufactured in-house)-covered Diamond stent (Boston Scientific, Watertown, MA, USA) had significantly lower occlusion rate and more long-term patency rate than those treated with an uncovered stent [3]. Similarly, Krokidis et al reported that in a prospective randomized study in 60 patients with malignant jaundice due to extrahepatic cholangiocarcinoma, a covered Viabil stent (W. L. Gore and Associates, Newark, DE, USA) inserted by percutaneous placement was patent for a significantly longer time and had lesser frequency of tumor ingrowth than uncovered Wallstents (Boston Scientific, Watertown, MA, USA) [13]. On the other hand, because the covered metallic mesh probably is not embedded in the bile duct epithelium, migration of the covered SEMS is a possible complication in malignant distal biliary obstruction, and migration occurs in 1.8% to 8.3% of the patients with covered SEMS and in 0% to 2.4% of patients with uncovered SEMS [3-7]. In addition, Kahaleh et al. reported that 80 patients with malignant biliary obstruction treated with covered Wallstents, early migration (within the first 30 days) was observed in 20% of the patients and late migration in 80% of the patients [5]. However, these migrations occurred through the papilla of Vater unlike that in our patient who showed migration through a different pathway.

The incidence of spontaneous internal biliary fistulas is 0.9% to 3.2% [14-16]. Choledochoduodenal fistulas usually occur as a complication of duodenal ulcerative disease, choledocholithiasis, trauma, or malignancies of the duodenum or biliary tract [14-18].

We thought that in our patient, the covered SEMS had migrated to the stomach through the choledochoduodenal fistula, which was present at the oral side of the obstruction of the descending part of the duodenum, by an enlargement in the pancreatic head tumor. Migration of the SEMS via a transpapillary pathway and subsequently through the duodenal obstruction is highly unlikely. The mechanism of formation of the choledochoduodenal fistula is mostly associated with a mechanical contact between the wall of the bile duct and the edge of the SEMS, which is pushed up in the direction of the duodenum because of the enlargement of the primary tumor, finally penetrating through the duodenal wall. The exact duration for which the SEMS was present in the fistula is unknown. However, the SEMS may not be present for a long period because jaundice was not present during the clinical course. As the one of the method to prevent the formation of a choledocoduodenal fistula by a biliary SEMS, the length of the first biliary SEMS might be as long as the extrahepatic bile duct in order to evade a bile duct wall and the physical contact with the edge of a biliary SEMS.

To our knowledge, the cases of only 3 patients with choledochoduodenal fistula due to SEMS have been reported thus far [8-10]. Ryozawa et al. reported the case of a patient with obstructive jaundice due to lymph node metastasis of rectal cancer; a choledochoduodenal fistula without stent migration was found 3 months after inserting uncovered SEMS because of tarry stool [8].In addition, Lee et al. reported a patient with ampulla of Vater carcinoma; a choledochoduodenal fistula without stent migration was diagnosed 40 days after placement of an uncovered metallic biliary stent because of a severe pain in the right upper quadrant of the abdomen with jaundice [10]. Krokidis et al reported a patient with cancer of the pancreatic uncinate process accompanied with late migration of a covered SEMS through a spontaneous choledochoduodenal fistula 13 months after stent insertion because of fatigue, weakness, and abdominal pain, and stent migration was not detected in the entire body [9]. Although Krokidis et al report that the stent migration occurred through the fistula, the actual route of stent migration remains to be clarified because the stent had already disappeared from the patient’s body at the time of the discovery. Therefore, the condition of our patient is extremely rare.

Owing to the characteristic of migration of covered SEMS, aggressive endoscopic removal of the SEMS is performed; the rate of complete removal of covered SEMS is 77.8% to 100% [19-21].

In conclusion, covered SEMS will increasingly be used in the palliative treatment of patients with malignant biliary obstruction. Close attention should be paid to rare adverse effects such as late migration through spontaneous choledochoduodenal fistula.

## References

[1] Davids PH, Groen AK, Rauws EA, Tytgat GN, Huibregtse K (1992). Randomised trial of self-expanding metal stents versus polyethylene stents for distal malignant biliary obstruction. Lancet.

[2] Kaassis M, Boyer J, Dumas R, Ponchon T, Coumaros D, Delcenserie R, Canard JM (2003). Plastic or metal stents for malignant stricture of the common bile duct? Results of a randomized prospective study. Gastrointest Endosc.

[3] Isayama H, Komatsu Y, Tsujino T, Sasahira N, Hirano K, Toda N, Nakai Y (2004). A prospective randomised study of "covered" versus "uncovered" diamond stents for the management of distal malignant biliary obstruction. Gut.

[4] Nakai Y, Isayama H, Komatsu Y, Tsujino T, Toda N, Sasahira N, Yamamoto N (2005). Efficacy and safety of the covered Wallstent in patients with distal malignant biliary obstruction. Gastrointest Endosc.

[5] Kahaleh M, Tokar J, Conaway MR, Brock A, Le T, Adams RB, Yeaton P (2005). Efficacy and complications of covered Wallstents in malignant distal biliary obstruction. Gastrointest Endosc.

[6] Yoon WJ, Lee JK, Lee KH, Lee WJ, Ryu JK, Kim YT, Yoon YB (2006). A comparison of covered and uncovered Wallstents for the management of distal malignant biliary obstruction. Gastrointest Endosc.

[7] Park do H, Kim MH, Choi JS, Lee SS, Seo DW, Kim JH, Han J (2006). Covered versus uncovered wallstent for malignant extrahepatic biliary obstruction: a cohort comparative analysis. Clin Gastroenterol Hepatol.

[8] Ryozawa S, Akiyama T, Ikeda M, Furui T, Yabushita Y, Kondo S, Nogichi T (1995). A case of cholangio-duodenal fistula formation after metallic stenting for malignant biliary stricture. Dig Endosc.

[9] Krokidis ME, Hatzidakis AA, Manousaki EG, Gourtsoyiannis NC (2008). Late migration of two covered biliary stents through a spontaneous bilioenteric fistula in a patient with malignant biliary obstruction. Cardiovasc Intervent Radiol.

[10] Lee TH, Park SH, Kim SP, Lee SH, Lee CK, Chung IK, Kim HS (2009). Spontaneous choledochoduodenal fistula after metallic biliary stent placement in a patient with ampulla of vater carcinoma. Gut Liver.

[11] Bethge N, Sommer A, Gross U, von Kleist D, Vakil N (1996). Human tissue responses to metal stents implanted in vivo for the palliation of malignant stenoses. Gastrointest Endosc.

[12] Hausegger KA, Kleinert R, Lammer J, Klein GE, Fluckiger F (1992). Malignant biliary obstruction: histologic findings after treatment with self-expandable stents. Radiology.

[13] Krokidis M, Fanelli F, Orgera G, Bezzi M, Passariello R, Hatzidakis A (2010). Percutaneous treatment of malignant jaundice due to extrahepatic cholangiocarcinoma: covered Viabil stent versus uncovered Wallstents. Cardiovasc Intervent Radiol.

[14] Zwemer FL, Coffin-Kwart VE, Conway MJ (1979). Biliary enteric fistulas. Management of 47 cases in native Americans. Am J Surg.

[15] Glenn F, Reed C, Grafe WR (1981). Biliary enteric fistula. Surg Gynecol Obstet.

[16] Yamashita H, Chijiiwa K, Ogawa Y, Kuroki S, Tanaka M (1997). The internal biliary fistula—reappraisal of incidence, type, diagnosis and management of 33 consecutive cases. HPB Surg.

[17] Griffith CD, Saunders JH (1982). Cholecystoduodenocolic fistula following abdominal trauma. Br J Surg.

[18] Shah P, Ramakantan R (1990). Choledochoduodenal fistula complicating duodenal ulcer disease (a report of 3 cases). J Postgrad Med.

[19] Familiari P, Bulajic M, Mutignani M, Lee LS, Spera G, Spada C, Tringali A (2005). Endoscopic removal of malfunctioning biliary self-expandable metallic stents. Gastrointest Endosc.

[20] Shin HP, Kim MH, Jung SW, Kim JC, Choi EK, Han J, Lee SS (2006). Endoscopic removal of biliary self-expandable metallic stents: a prospective study. Endoscopy.

[21] Ishii K, Itoi T, Sofuni A, Itokawa F, Tsuchiya T, Kurihara T, Tsuji S (2011). Endoscopic removal and trimming of distal self-expandable metallic biliary stents. World J Gastroenterol.

